# Rising Voltage-Gated Potassium Channel Antibody Level as a Possible Disease Progression Marker for Amyotrophic Lateral Sclerosis: A Case Report

**DOI:** 10.7759/cureus.76760

**Published:** 2025-01-01

**Authors:** Sophia Salter, Ethan Salter, Aengela Jihyoun Kim, Antonio K Liu

**Affiliations:** 1 Neurology, Adventist Health White Memorial, Los Angeles, USA; 2 Internal Medicine, Adventist Health White Memorial, Los Angeles, USA; 3 Neurology, Loma Linda University School of Medicine, Loma Linda, USA

**Keywords:** amyotrophic lateral sclerosis, autoimmune disease, disease marker, immune modulation therapy, voltage gated potassium channel antibody

## Abstract

A subset of amyotrophic lateral sclerosis (ALS) patients tests positive for antibodies commonly associated with autoimmune neurological diseases, including voltage-gated potassium channel (VGKC)-complex antibodies. Although an autoimmune basis for ALS remains speculative, and immunomodulatory therapies have shown minimal benefit as of yet, isolated cases suggest that VGKC-complex antibodies may be relevant to disease type and progression. In this report, we present a case of ALS in which increasing VGKC-complex antibody levels correlated with clinical decline, raising the question of whether such antibodies could serve as biomarkers of progression in VGKC-complex antibody-positive ALS patients. To date, no published studies have systematically evaluated changes in VGKC-complex antibody levels in ALS patients over time. Our findings suggest that tracking VGKC-complex antibodies in ALS may offer insights into disease progression and prompt further investigation into their potential role as prognostic biomarkers, especially in certain subtypes of the disease.

## Introduction

Amyotrophic lateral sclerosis (ALS), also known as Lou Gehrig’s disease, is a progressive multisystem neurodegenerative disease characterized by the degeneration and death of motor neurons in the brain and spinal cord. These motor neurons control almost all parts of the body including the arms, legs, chest, throat, and mouth. As the motor neurons degenerate, the associated muscles weaken, leading to paralysis and ultimately death. Historically, causes of ALS have been determined to be both genetic and environmental in nature, although ALS caused by environmental factors, or sporadic ALS, is much more common, occurring in 90% of cases [1]. In the 10% of genetically-based ALS cases, while more than 20 genes contributing to ALS have been identified, the most common genetic causes result from hexanucleotide repeat expansions in the *C9orf72* gene and mutations in *SOD1*,* TARDBP*,* FUS*, and *TBK1* [2].

ALS onset typically ranges from 40 to 70 years of age, beginning with weakness in the distal muscles and spreading across the body as the disease progresses. Alongside muscle weakness, 35-40% of patients experience mild cognitive or behavioral changes, and 10-15% experience frontotemporal dementia which is most common in individuals with the *C9orf72* mutation [2]. On average, the time from symptom onset to diagnosis is 11 months, and once diagnosed with ALS, patients generally live three to five years before they experience respiratory failure and then death [3]. While there are several medications demonstrated to slow disease progression depending on ALS type, primarily tofersen, edaravone, and riluzole, none of these have demonstrated the ability to halt disease progression. Riluzole, for example, only extends survival without ventilator dependence by two to three months [4]. Given the limitations of current treatments, further research into ALS's etiology and pathogenesis is crucial for developing new therapeutic approaches.

More recent studies on ALS have indicated that the immune system plays a large role in pathogenesis. As ALS progresses, the central and peripheral immune systems are activated, leading to a chronic proinflammatory microenvironment [5]. At this time, there is no clear consensus on exactly how the immune system is involved in the pathogenesis of ALS. Some data have found that in ALS, there is an increase in neurotoxic Th1/M1 cells and fewer neuroprotective Tregs/M2 cells in the central nervous system, and this ratio may be associated with disease progression [6]. There are also studies being done on the peripheral nervous system. Some studies highlight systemic immune activation, including increased neutrophils, monocytes, and inflammatory dendritic cells, as well as a reduction in regulatory T cells [7]. There is also some evidence to suggest Schwann cells may contribute to the pathogenesis of ALS by modulating immune responses through the expression of MHC molecules, which can alter T cell recruitment and affect neurodegeneration [8]. Additionally, Schwann cells may impact neuromuscular junction degeneration through immune responses, such as through mast cell infiltration [9].

Studies on the serum and cerebrospinal fluid (CSF) from ALS patients have found elevated levels of numerous paraneoplastic and neuron-related antibodies [5]. Related studies have found that these antibodies may have a significant impact on ALS progression. For example, when IgG antibodies purified from the serum of patients with ALS were injected into mice, the mice exhibited ALS symptoms, including loss of spinal motor neurons and muscle weakness in the limbs [10].

Alongside understanding the roles these antibodies play in disease progression, several antibodies are currently being investigated as potential biomarkers for monitoring ALS progression. Among these, VGKC-complex antibodies are of particular interest. VGKCs play a large role in membrane excitability regulation. Several immune-mediated diseases are associated with VGKC-complex antibodies, including Morvan’s syndrome, peripheral nerve hyperexcitability (PNH), and limbic encephalitis (LE) [11]. In general, VGKC-complex antibody levels are considered clinically relevant if they are greater than 100 pmol/l. However, these levels can vary greatly depending on the disease in question. For example, while patients with LE typically present with VGKC-complex antibody levels greater than 400 pmol/L, patients with PNH commonly present with VGKC-complex antibody levels between 100 pmol/L and 400 pmolk/L [11].

Due to the usual rapid functional decline and death in ALS patients, the discovery of accurate prognostic biomarkers can be critical for tracking disease progression and therapeutic efficacy, and also in stratifying patients. However, currently, though there are some hypothesized biomarkers, many are debatable and none have achieved being clinically and routinely applicable [12]. An ideal biomarker for ALS would be stable in the fluid, sensitive to ongoing injury, and possibly specific to the underlying pathology. One major reason for the lack of universal biomarkers for ALS that serve clinical utility in diagnostic and more importantly prognostic function is the many subtypes of ALS with their variability in pathologies and chemical markers. It may be, at this time, more achievable to find prognostic biomarkers that apply to specific subtypes of ALS [13].

In this report, we present a case of ALS with positive VGKC-complex antibody levels (>100 pmol/L) that increased with disease progression. We hope that by examining the patient’s medical history, neurological presentation, and VGKC-complex antibody levels, we can contribute to a deeper understanding of VGKC-complex antibody levels in ALS and their potential for use as biomarkers in ALS progression monitoring.

## Case presentation

A 53-year-old male patient presented to our emergency department, and later was admitted, with worsening choking and difficulty swallowing. He was initially unable to provide much about his medical history. His medical record which contained detailed information on his diagnosis/management from a regional university neuromuscular clinic was not initially or immediately available. On exam, the patient exhibited both upper and lower motor neuron findings. He had retained extraocular movements, tongue fasciculations, dysarthria, dysphonia, and dysphagia. He had diffused muscle atrophy in all four extremities. Crossed adductor reflex and Hoffman’s and Babinski’s signs were all present. Non-sustained clonus was observed in both ankles. The patient was unable to stand without assistance.

Since his previous medical records were not available, further studies were performed including CSF analysis, ganglioside antibodies, acetylcholine receptor, and related antibodies, as well as an autoimmune neurological diseases antibody panel. CSF analysis revealed 1 WBC/mm^3^, protein at 72 mg/dL (upper limit 45), and myelin basic protein (MBP) mildly elevated at 6.13 ng/mL (0-.5.5) (Table 1). CT scans of the head and spine were unremarkable. MRI could not be obtained due to bullet fragments in the patient’s body. Medical records from a university neuromuscular clinic later became available which revealed he was given the diagnosis of definite ALS according to the El Escorial revised criteria. His symptom onset was about 24 months prior to this current admission and he had already been started on riluzole. The copper-zinc (Cu/Zn) superoxide dismutase mutation was negative. Electromyography (EMG) and nerve conduction studies (NCS) were performed which yielded results consistent with ALS. His ALS Functional Rating Scale (ALSFRS) at the time of this current admission was 30. Among all the antibodies sent, the only one that came back positive was for the VGKC-complex antibody at 242 pmol/L (0-31). Solu-medrol and two courses of intravenous immunoglobulin (IVIG) were administered over the next two months with no obvious benefit. At a six-month follow-up (30 months after the start of initial symptoms), he had an ALSFRS score of 26 and his VGKC-complex antibody level had risen to 279 pmol/L (Table 1).

**Table 1:** Laboratory values and results CSF: cerebral spinal fluid; VGKC: voltage-gated potassium channel; ALSFRS: Amyotrophic Lateral Sclerosis Functional Rating Scale

Results	Patient values	Normal Range
CSF WBC	1 WBC/mm^3^	0-5 WBC/mm^3^
CSF protein	72 mg/dL	0-45 mg/dL
CSF Myelin Basic Protein	6.13 ng/mL	0-5.5 ng/mL
VGKC antibody initial	242 pmol/L	0-31 pmol/L
VGKC antibody at the six-month follow-up	279 pmol/L	0-31 pmol/L
ALSFRS score initial	30	-
ALSFRS score at the six-month follow-up	26	-
VGKC antibody increase rate	6.17 pmol/L/month	-
ALSFRS score decrease rate	0.67 per month	-
VGKC vs ALSFRS relative rate change	9.25 pmol/L increase for each ALSFRS point dropped	-

## Discussion

Our diagnostic workup of this patient, as well as previous evaluations at tertiary centers, firmly supported a diagnosis of definite ALS. However, some unique aspects of this case are worth noting: the presence of VGKC-complex antibodies, which increased from 242 to 279 pmol/L, a 37 pmol/L rise over six months, as the patient’s ALSFRS score declined by 4 points in the same period. Additionally, there was a mild elevation in CSF MBP in the patient. As seen in Table 1, the VGKC antibody level increased by 6.17 pmol/L each month, the ALSFRS score decreased by 0.67 per month, and for each ALSFRS point dropped, the VGKC antibody level rose by 9.25 pmol/L.

Of note, some studies have reported CSF MBP as being elevated in ALS, especially in those patients with frontotemporal dementia [14]. Blood-brain barrier dysfunction was brought into the spotlight as part of the pathologic deterioration of ALS. Our patient indeed had a mild CSF MBP elevation, but our study was not able to observe the changes in MBP levels.

Though the presence of autoimmune antibodies in ALS is uncommon, there have been cases reported. There is no current evidence that VGKC-complex antibodies are pathogenic to ALS, but their significance is still being explored. A recent 2024 narrative review by Liu et al. discussed 28 different antibodies among 121 studies, with four of these having a major focus on VGKC-complex antibodies in ALS [5]. These four studies collectively observed VGKC-complex antibodies in 33 ALS patients [15,16,17,18]. While some authors attempted immunomodulation treatments, only one study documented any improvement, and even then, it was anecdotal, transient, and modest. Almost all of these studies reached a similar conclusion: the presence of VGKC-complex antibodies, in general, does not seem to affect ALS progression or outcomes. For example, a case-control study comparing motor neuron disease (MND) patients with and without cation channel antibodies, including VGKC patients, found that only 6.9% of MND patients had positive cation channel antibodies, typically at low levels, with no effect on disease progression [16]. It is important to note that this study was for MNDs in general, with ALS being a type of MND.

However, another study reported higher VGKC-complex antibody levels in certain ALS subsets, and that VGKC-complex antibodies were more frequent in ALS than in peripheral nerve disorders [17]. In a different study, Godani et al. found VGKC-complex antibodies to be more common in slow-progressing ALS cases, where ALSFRS declines were fewer than 3 points over nine months. They also hypothesized that these antibodies could emerge as an immune response to nervous system damage, and proposed VGKC-complex antibodies as a possible prognostic marker for certain subtypes of ALS [18]. A study, not part of Liu et al.'s [5] narrative review, found an ALS patient with prominent cramps and fasciculations as having a very high VGKC-complex antibody level of 907.5 pmol/L [19]. This patient died after two years of symptom onset from respiratory failure; an autopsy performed on the patient found degeneration of lower motor neurons being predominant and out of proportion compared to that of upper motor neurons. Another study noted reduced axonal potassium channel expression in human sporadic ALS, suggesting that resulting axonal hyperexcitability could contribute to increased fasciculations and motor neuron death [20]. Therefore, these studies seem to show some patterns and associations with VGKC-complex antibodies with different patterns or subsets of the disease.

ALS, being a heterogeneous disorder, includes both genetic and sporadic forms, and manifests as different clinical phenotypes with varied progression rates such as patterns of upper vs lower motor neuron predominance, bulbar dominance, and even frontotemporal features. Therefore, at this point in time, it may be difficult to find prognostic biomarkers that are generally applicable to ALS, and this discrepancy in the association with ALS and VGKC-complex antibodies may be partially due to the variable nature of ALS.

As is the variability of ALS subtypes and pathologies, there are also many different types of VGKC with varying functions. For example, VGKC Kv1.3 is ubiquitously expressed on plasma membranes of T and B lymphocytes, macrophages, fibroblasts, and more. Activation of Kv1.3 has been associated with neurodegenerative diseases. One study used SOD1 mutation mice who had reduced motor function and found that inhibiting Kv1.3 improved these motor deficits and increased survival time for SOD1 mice [21]. There were both functional and chemical changes observed in these mice. In another study, the downregulation of Kv8.1, which is considered a “silent” channel and serves to interact with Kv2 to change its properties, increased motor neuron variability in SOD1 ALS patients. Kv8.1 was studied to be decreased in the motor cortex of ALS patients of sporadic type [22]. In another study, human sporadic ALS showed reduced axonal potassium channel expression [20]. Kv1.2 was observed to be reduced in ventral roots but normal in dorsal roots of ALS patients from autopsies of five different cases and it was proposed that the decreased potassium currents in ALS patients perhaps was associated with hyperexcitability and fasciculations in ALS patients. Therefore, there may also be utility in studying even specific types of VGKC antibodies along with specific subtypes of ALS. The complexity of VGKC with its types and ALS subtypes presents much to explore further.

In summary, the recent study on the clinical significance of various antibodies in ALS [5] and other studies suggest that the presence of VGKC-complex antibodies may indicate the presence of ALS subtypes such as those with a characteristic survival time and a possible correlation with disease progression. However, these studies have only reported a single value for each patient, and have not followed the VGKC antibody levels over time. Our case presents a unique observation of progressively increasing VGKC-complex antibody levels, which appear to correlate with symptomatic worsening and a decline in ALSFRS scores, offering a potential avenue for longitudinal biomarker research in ALS. In our case, the VGKC antibody value increased by 9.25 pmol/L for each ALSFRS score lost.

## Conclusions

This case suggests that rising VGKC-complex antibody levels may correlate with ALS disease progression in patients who test positive for these antibodies. Although the autoimmune component of ALS remains speculative, our findings highlight the potential role of VGKC-complex antibodies as a biomarker for tracking disease advancement, particularly in certain ALS subtypes. The variability in VGKC antibody levels and types across patients with ALS suggests that some may have distinct disease trajectories, patterns, or underlying pathologies. The limitation of our report is that it involved a single case only. Overall, the rarity of detecting autoimmune antibodies among ALS patients makes analysis challenging. Further research into VGKC-complex antibodies in ALS, including their potential prognostic value and pathophysiological role, may provide greater insights into disease mechanisms and contribute to more personalized approaches in ALS care. Controlled studies focused on antibody levels over time could determine if tracking VGKC-complex antibodies can inform clinicians on ALS progression, potentially leading to novel therapeutic strategies tailored to immune-modulated ALS subtypes.
